# Heavy metal whole-cell biosensors using eukaryotic microorganisms: an updated critical review

**DOI:** 10.3389/fmicb.2015.00048

**Published:** 2015-02-20

**Authors:** Juan C. Gutiérrez, Francisco Amaro, Ana Martín-González

**Affiliations:** Departamento de Microbiología-III, Facultad de Biología, Universidad Complutense, Madrid, Spain

**Keywords:** biosensors, heavy metals, eukaryotic microorganisms, bioassays, yeasts, microalgae, ciliates

## Abstract

This review analyzes the advantages and disadvantages of using eukaryotic microorganisms to design whole-cell biosensors (WCBs) for monitoring environmental heavy metal pollution in soil or aquatic habitats. Basic considerations for designing a eukaryotic WCB are also shown. A comparative analysis of the promoter genes used to design WCBs is carried out, and the sensitivity and reproducibility of the main reporter genes used is also reviewed. Three main eukaryotic taxonomic groups are considered: yeasts, microalgae, and ciliated protozoa. Models that have been widely analyzed as potential WCBs are the *Saccharomyces cerevisiae* model among yeasts, the *Tetrahymena thermophila* model for ciliates and *Chlamydomonas* model for microalgae. The advantages and disadvantages of each microbial group are discussed, and a ranking of sensitivity to the same type of metal pollutant from reported eukaryotic WCBs is also shown. General conclusions and possible future developments of eukaryotic WCBs are reported.

## INTRODUCTION

Metals are important elements in living systems, because several of them act as essential cofactors for many enzymes involved in cellular metabolism and growth. Without these essential metals, there would be no life. On the other hand, certain metals (mainly those considered as “heavy metals”) are among the most abundant, toxic and persistent inorganic environmental pollutants (Hill, 2004). Mining and other industrial anthropogenic activities have increased the heavy metal content in both terrestrial and aquatic ecosystems. Unlike complex organic pollutants, they cannot be degraded by microorganisms, although they can be inactivated and accumulated by both prokaryotic and eukaryotic cells (Martin-Gonzalez et al., 1999). Almost all heavy metals have toxic effects on organisms and can cause important ecological disturbances. For instance, metals can, directly or indirectly, produce reactive oxygen species (ROS), and originate significant alterations in proteins, nucleic acids, and lipids (Leonard et al., 2004; Valko et al., 2005), which can induce cell death by necrosis or apoptosis (Pulido and Parrish, 2003). Furthermore, certain metals are involved in human carcinogenesis (Valko et al., 2006). Because of the ecological, sanitary, and economic consequences of heavy metal pollution, several of these metals are considered to be priority environmental pollutants.

**FIGURE 1:**
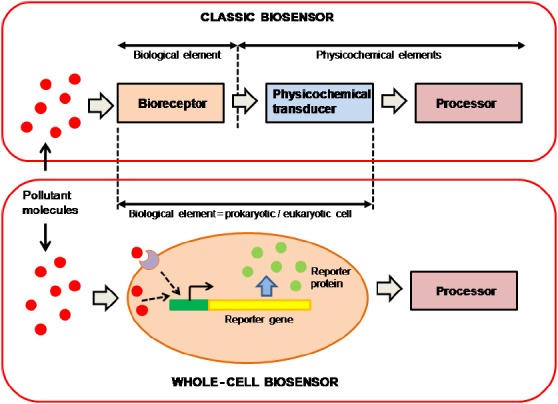
**Schematic representation of classic and whole-cell biosensor elements**.

It is difficult to predict the global effects of the increase in different types of environmental pollutants, so there is an overriding need to develop screening methods for environmental monitoring. While metal concentrations can be measured using molecular recognition or chemical analysis, critical parameters such as bioavailability, toxicity, and genotoxicity, can only be assayed using living cells. The most sensitive screening methods for detecting pollutants are those that incorporate biological components that are used as targets for an active substance or pollutant. In general, these are known as biosensors or bioreporters. The classic biosensor can be defined as an integrated bioreceptor-physicochemical transducer device. A classic biosensor consists of three different elements: a bioreceptor or biological recognition element, which interacts with the pollutant molecules, a physicochemical transducer, which converts the biological response into a detectable physicochemical signal, and a microelectronic processor of this signal, which amplifies it and converts it into a numeric record (Figure 1). The biological component of these devices may be microorganisms or whole-cells, tissues, organelles, cell receptors, enzymes, or specific proteins, nucleic acids, or antibodies. With regard to the transducer, at least four different types of transduction element can be considered: electrochemical, optical, piezoelectric, or thermometric. Construction of these biosensors requires biological and physicochemical knowledge. This involves interdisciplinary cooperation between different specialists, which makes construction more difficult and expensive.

More recently, several authors have introduced the concept of the whole-cell biosensor (WCB) as a very useful alternative to classical biosensors (Belkin, 2003; Van der Meer and Belkin, 2010). A WCB uses the whole prokaryotic or eukaryotic cell as a single reporter, incorporating both bioreceptor and transducer elements into the same cell (Figure 1). Organisms used as WCBs are generally experimentally modified to incorporate transducer capacity or increase their sensitivity.

**FIGURE 2:**
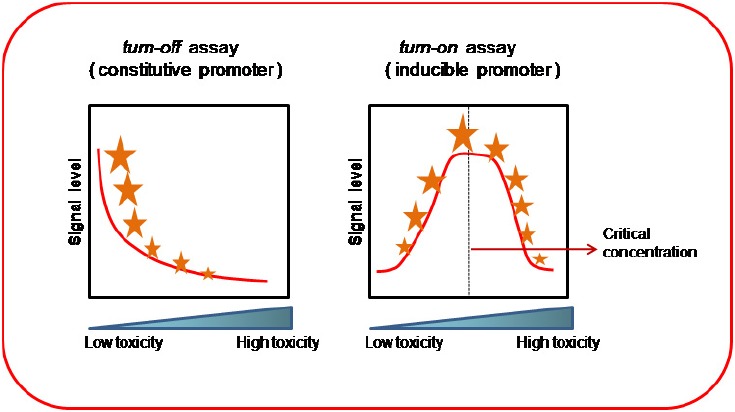
***Turn off* and *turn on* bioassays**. *Turn off* assays use constitutive promoters; the signal level from the reporter gene decreases proportionally to toxic pollutant concentration. *Turn on* assays use inducible promoters; the reporter signal level increases with pollutant concentration. This reporter signal may reach a maximum value (critical concentration), after which decreases due to the toxic effect on the cell. The critical concentration value will depend on the degree of cellular resistance to the pollutant.

Two types of bioassays can be considered when using WCBs: *turn off* and *turn on* assays (Belkin, 2003). *Turn off* assays are similar to general microbial toxicological bioassays; the sample toxicity is estimated from the degree of inhibition of a cellular activity (e.g., growth inhibition, respiration, motility depletion, etc.), or a specific reporter gene expression. In these bioassays, the toxic concentration is proportional to the measurement of any cellular function inhibition (Figure 2). In *turn on* assays, however, a quantifiable molecular reporter is fused to a specific gene promoter, known to be activated by the chemical or environmental pollutant. Therefore, the sample toxicity is proportional to the gene expression of the reporter molecule (Figure 2).

These screening methods can be applied to detect the presence of any environmental pollutant causing general stress in cells or organisms present in the ecosystem, or a specific pollutant such as certain heavy metals. *Turn off* assays are more unspecific, because the signal decreases as a result of a broad range of cytotoxic effects, while *turn on* assays, based on an inducible gene expression, are usually more specific, as induction of the gene reporter only takes place when the pollutant is present. Their specificity will therefore depend on the degree of the gene promoter specificity to be opened by an exclusive pollutant of a chemically related group of pollutants. With respect to specificity, WCBs can be divided into effect- and compound-specific sensors (Yagi, 2007). Effect-specific biosensors are stimulated by changes in a physicochemical condition (e.g., pH, temperature, or osmotic changes) or pollutants that give rise to a specific type of toxicity (e.g., oxidative stress or protein damage). Compound-specific biosensors respond to only one type of pollutant or compounds with similar chemical features (e.g., any heavy metal). Other specificity-based classifications of WCBs are: (i) class-I biosensors that only respond to a specific or exclusive pollutant increasing the signal, (ii) class-II biosensors that respond to a specific cellular stress increasing the signal, and (iii) class-III biosensors that respond unspecifically to different pollutants or stress conditions.

With regard to the cellular type of WCB for detecting metals, about 85% of these are based on genetically modified bacteria (Magrisso et al., 2008), while ∼15% are based on eukaryotes. Of the 15% of eukaryotes biosensors, the majority of microorganisms are yeasts. In this review, only eukaryotic WCBs (including yeasts, microalgae, and protozoa) are considered. The advantages and disadvantages of these three types of eukaryotic microorganisms as heavy metal WCBs are discussed and compared. Likewise, we discuss on the different potential reporter molecules to be selected as the WCB transducer element.

## REASONS FOR USING EUKARYOTIC MICROORGANISMS AS WCBs

There are several good reasons for using eukaryotic microorganisms as WCBs. In the first place, the use of “microorganisms,” prokaryotic or eukaryotic, is an advantage in itself. In order to get a sufficiently quantifiable signal from the WCB, a good level of biomass must be reached in a short time. This can be obtained using organisms with a high growth speed or short generation time, features that are almost exclusive to microorganisms. Another advantage of using microorganisms as WCBs is that most of them can be easily manipulated and grown on a wide variety of different media or culture types. Likewise, big advances in microbial genetic analysis methodology and the increase in genomic sequencing make the experimental modifications needed for introducing transduction capacity during WCB design easier. Furthermore, microorganisms are distributed all over the world, and occupy all known ecosystems, which constitutes a great advantage when the specialist is looking for particular microbial capacities for designing a specific WCB to detect a specific environmental pollutant.

Another advantage of “eukaryotic” microorganism is the possibility of using cells from three different taxonomic groups: fungi, microalgae, or protozoa. The “eukaryotic” characteristic is particularly important because, in general, WCBs try to detect potential environmental toxic substances for other eukaryotic organisms (including humans). The existence of a more similar metabolism, genome, and cellular organization in these eukaryotic biosensors with those organisms undergoing chemical pollution, makes the extrapolation and comparison of results more accurate and reliable.

## CONSTITUTIVE VERSUS INDUCIBLE EXPRESSION SYSTEMS

A constitutive expression system generally uses a gene promoter that is highly expressed under normal conditions, which results in a high basal expression level of the reporter gene. During exposure of the WCB to a potential toxic pollutant, the basal expression level is decreased due to the toxic effect, so reduction in this parameter is correlated and inversely proportional to the environmental sample toxicity (Figure 2). *Turn off* bioassays are based on this type of WCB, using gene constructs with constitutive promoters. Therefore, a constitutive WCB shows a general view of the degree of toxicity of the polluted sample based on unspecific metabolic alterations. This affects the general gene expression of the cell, slowing the growth rate, or inducing cell mortality and reducing the intensity of the reporter signal. Generally, the promoters selected for designing these WCBs are those regulating the gene expression of housekeeping genes, such as ribosomal proteins, cell structural proteins (e.g., tubulin, actin, etc.), or certain metabolism enzymes, which maintain gene expression at a sufficiently high and constant level.

The information given by constitutive systems offer applies only to the general toxicity of the sample or stress, and not to the nature of the toxic element, pollutant, or stressor. A constitutive WCB is included in class-III biosensors. Although these are not specific biosensors, they are useful for detecting the presence of toxicity or general stressors in an ecosystem.

Inducible systems use constructs which fuse an inducible promoter to a reporter gene. To design an inducible WCB, a specific promoter, which is only opened by a specific pollutant (or group of related pollutants), is selected to carry out the gene construct. In general, the gene expression level regulated by these types of inducible promoters is high, because the gene product originated may constitute an important part of the cellular defense against a particular stressor or toxic substance (Figure 2). Selection of the ideal promoter to be used in the design of a WCB for detecting a specific pollutant requires prior analysis of the transcriptional capacities of the cell under that specific pollutant. Transcriptional data can be obtained from a cDNA gene-library construction or by using a microarray with RNA populations isolated from cells previously treated with the pollutant or stressor. These WCBs are classified as class-I biosensors, and present considerable specificity because they respond almost exclusively to one type of pollutant or stressor. They are very useful for monitoring specific environmental pollutants, but are more difficult to construct because there are very few gene promoters that respond exclusively to one type of compound.

## SELECTING THE REPORTER GENE

Another important element for designing any WCB is the selection of a reporter gene, with the transducer function that will convert the biological response into a detectable physicochemical signal. The most commonly used reporters, applied to eukaryotic microorganisms can be classified into two groups (Robbens et al., 2005): (i) substrate-dependent reporters, and (ii) substrate-independent reporters or fluorescent proteins. Included in the first group are those reporters that are based on prokaryotic β-galactosidase enzymatic activity (adapted to eukaryotic cells) and eukaryotic luciferases. The gene encoding β-galactosidase (*lacZ* gene from the *Escherichia coli* lactose operon) is involved in the cleavage of the disaccharide lactose (galactose + glucose) but this enzyme can also use other substrates (related to lactose) that have been used to register gene expression. One of these is *o*-nitrophenol-β-D-galactopyranoside (ONPG), which releases galactose and *o*-nitrophenol (yellow in color) after it is broken up by the β-galactosidase. The appearance of a yellow color reveals the presence of β-galactosidase activity and the gene expression of the *lacZ* reporter gene. The colorimetric reaction has a sensitivity level (or β-galactosidase detection limit) of about 100 pg, and can determine differences between the expression levels of a specific promoter under different conditions when it is fused with the *lacZ* reporter gene. Greater sensitivity (up to 12 fg) is obtained by using other substrates and fluorimetric analysis, such as methyllumbelliferyl-β-D-galactopyranoside (MUG) or fluorescein-di-β-D-galactopyranoside (FDG), which (after β-galactosidase activity) release free umbelliferone (a strongly fluorogenic compound) or fluorescein, respectively. The main problem with these fluorogenic reporter systems is their low permeability or penetration of the respective substrates into the cell; WCBs therefore, have to be lysed in order to measure the enzymatic activity or reporter gene expression.

With regard to eukaryotic luciferases, one of the most commonly used as a bioluminescent reporter is that of the firefly (*Photinus pyralis*; Gu et al., 2004). This enzyme catalyzes a two-step oxidation reaction (Greer and Szalay, 2002): the substrate luciferin (a benzothiazole) is converted to luciferyl-adenylate (active form), using ATP as a co-factor, and is then oxidated to oxiluciferin, emitting light (at 550–570 nm) that can be quantified by a luminometer. Likewise, addition of the substrate (luciferin) to the cell lysate is necessary for carrying out the bioassay. The addition of coenzyme A stabilizes the luminiscence signal for several minutes (Greer and Szalay, 2002). The sensitivity level or detectable limit of this reporter gene is at subattomole concentrations (<10^–18^ mol) and it also shows a linear response with regard to the concentration.

All these substrate-dependent reporters require substrate addition and cellular lysis (with the exception of one type of eukaryotic cells), which increases the laboriousness, the number of manipulation errors, the cost and the complexity of the bioassays. However, these bioassays (i.e., the luciferase–luciferin system) are among the most sensitive.

Reporter genes encoding fluorescent proteins are substrate independent reporters, and obtain an immediate signal without substrate addition and cellular lysis. The most popular and widely used fluorescent protein is the green fluorescent protein (GFP) isolated from the Pacific jellyfish *Aequoria victoria*. This intrinsically fluorescent protein can be heterologously expressed in both prokaryotic and eukaryotic systems, and has been selected as a reporter gene for many experiments, especially those using eukaryotic cells. A great advantage of using this reporter protein in bioassays is that there is no need to add exogenous substrates or cofactors to measure fluorescence. GFP is non-invasive and can be detected (by fluorescence microscopy) and quantified (by flow cytometry) in complete cells. However, GFP requires a longer time to get a stable fluorescence emission, and more time is needed to detect differences among the expression levels of different samples. On the other hand, once fluorescence stability is reached, it continues for a prolonged time, which might be a problem in toxicology studies where cell mortality occurs (biosensors with a constitutive system), because the GFP continues to fluoresce after the cells have died. Table 1 shows the advantages and disadvantages for each reporter gene used in eukaryotic WCBs.

**Table 1:** **Comparison of the advantages and disadvantages of different reporter genes used in WCBs**.

**Reporter gene**	**Advantages**	**Disadvantages**
β-Galactosidase(*lacZ*)	Good stability Sensitivity depending on substrate.	Substrate dependent Low permeability
	No ATP requirement	Cellular lysis requirement
Eukaryotic luciferase(*luc*)	Rapid response	Substrate dependent
	Very high sensitivity	O_2_ and ATP requirement Low permeability and stability Cellular lysis requirement^1^
Green Fluorescent Protein (*gfp*)	Good stability	Moderate sensitivity
	Substrate independent No ATP requirement	Lag-time for stable fluorescence. Fluorescence after cell death
	No cellular lysis	Autofluorescence background

1 Not necessary in ciliates.

## DESIGNING AN EUKARYOTIC HEAVY METAL WCBs

On the basis of that has already been indicated, the eukaryotic whole cell to be selected as a WCB for heavy metal detection should have a high sensitivity to these inorganic pollutants. In general, cells that are highly sensitive to metals respond rapidly and intensely to metal stress. The best way to know the molecular basis of this response is to select the gene promoter to be used in the WCB construction. These gene promoters are usually elements of an inducible system, and respond strongly and specifically to metals. These genes constitute the first cellular defense against metals, and habitually correspond to metallic ion chelating molecules. Three main chelating molecules are involved in the cellular response to metals: glutathione (a tripeptide), phytochelatins (oligopeptides), and metallothioneins (proteins; Gutiérrez et al., 2008). Promoters from genes encoding enzymes acting in glutathione or phytochelatin biosynthesis and those in the ribosomal synthesis of metallothioneins may both be good candidates when selecting strong inducible promoters to be used in heavy metal WCBs. Because metals may induce ROS, another option is to select gene promoters regulating the expression of antioxidant enzymes, such as superoxide dismutases, catalases, glutathione peroxidases, glutathione, and thioredoxin reductases, among others. Several of these genes are strongly induced by metals in eukaryotic microorganisms (Gutiérrez et al., 2008).

After selecting the type of eukaryotic microorganism for designing the WCB and the inducible gene promoter for carrying out the gene construct, the final step is to select the reporter gene. The two main reporter systems that are the best options for eukaryotic WCBs are: the eukaryotic luciferase or the GFP. Selection of one or the other will depend on the requirements which may be greater sensitivity, higher stability, or a substrate-independent reporter (Table 1). Finally, the WCB should be extensively tested with several different heavy metals (including metal mixtures) under different conditions (metal concentration, culture medium, or buffer composition, pH, temperature, etc.) and the chemo-luminescence or fluorescence signal emission by the WCB quantified and evaluated. Likewise, a final assessment of the WCB should be carried out using real environmental terrestrial or aquatic samples. This evaluation is of great importance for knowing whether or not the WCB is useful for detecting the presence of bioavailable metals in real complex environmental samples. However, this last decisive step is not usually carried out by the majority of researchers, who report exclusively experimental data obtained from using samples prepared in the laboratory. After all the bioassays are performed, the new WCB is ready to be applied to environmental metal pollution monitoring in the real world.

As well as the previous considerations, other general features and requirements must be taken into account for designing new heavy metal WCB, for example: (i) good specificity, or inclusion as class-I or -II biosensors; these respond specifically and exclusively to a particular metal or metal stress; (ii) good sensitivity, or with a minimum detectable metal concentration at or below the maximum allowable concentration of each heavy metal, as established by the European Directive (86/278/CEE), for soil, or by the World Health Organization for drinking water; (iii) reproducible results over time among different polluted samples; (iv) a rapid response (less than 1 h); (v) ease of use, with a simple methodology to detect/quantify the metal in the sample; and (vi) longevity or stability (the biosensor must maintain sensor capacity during the whole monitoring test).

## YEASTS AS HEAVY METAL WCBs

Yeasts are well-known eukaryotic microbial models that are widely used in toxicology and basic biological studies. Among them, *Saccharomyces cerevisiae* is the most widely used eukaryotic microorganism in biotechnology and bioengineering, and some authors (Walmsley and Keenan, 2000) consider that it has certain advantages as a biosensor when exposed to the real world. For instance, it is a robust microorganism with a high physicochemical tolerance and good genetic tools that make possible the construction of genetically engineered yeasts with properties that are optimized for being better biosensors.

However, like prokaryotes, these have a cell wall that protects the cell and acts as a selective barrier to the entry of very different molecules (including substrates used by the biosensor transducer system), which makes transducer signal emission more difficult. Therefore, it is necessary to increase cell wall permeability before using them as WCBs, which constitutes an additional difficulty. Mutants with enhanced cell permeability can be used for this purpose (Terziyska et al., 2000; Walmsley and Keenan, 2000).

Despite the advantages shown for these eukaryotic microorganisms, few WCBs have been designed using yeasts, and, as occurs with bacteria, they have been used almost exclusively as the bioreceptor element in the construction of classic biosensors (Baronian, 2004). Therefore, only a few cases of real WCBs using yeasts can be described.

A WCB using *S. cerevisiae* cells and GFP as the reporter protein was developed to detect copper ions (Shetty et al., 2004), using the transcriptional activator protein Ace1 present in this yeast to control expression of the reporter gene *gfp*. When Cu ions are present in the sample, the AC1 protein activates the *cup1* promoter located upstream from the *gfp* gene, thereby inducing GFP production. This construct (*P_cup1_::gfp*) is included in a plasmid. This system can detect Cu^2+^ at concentrations as low as 0.5 μM, and is selective for Cu^2+^ over other metals (except for Ag^2+^; Shetty et al., 2004). Another similar *S. cerevisiae* WCB for Cu^2+^ detection has been constructed using the same promoter (*cup1*) but a different reporter gene (luciferase), with a similar limit of detection of about 0.5 μM, for Cu^2+^ ions was reported (Roda et al., 2011).

From a microarray gene expression analysis, under Cd^2+^ treatment, of the methylotrophic yeast *Hansenula polymorpha*, several over-expressed genes were selected. A comparative analysis of these revealed that the promoter from the *SEO1* gene (with an unknown cellular function), fused with the GFP gene, was the reporter construct with the highest GFP expression level with regard to other promoters tested (Park et al., 2007). The limit of detection for Cd^2+^ using this reporter system was about 1 μM, and is not specific for Cd^2+^ because it is also inducible by As^3+^, while the *SEO1* promoter from *S. cerevisiae* revealed that this is inducible by As^3+^ > Cd^2+^ > Hg^2+^, being, likewise, unspecific for cadmium.

## MICROALGAE AS HEAVY METAL WCBs

WCBs based on microalgae are much scantier. These photosynthetic microorganisms have been used as toxicity reporters in many classic biosensors (with a physicochemical transducer) because of their sensitivity to pesticides and metals. In general, these microorganisms constitute the bioreceptor element of the classic biosensor as an integrated whole cell sensor.

Microalgae are important in biosensor construction for marine applications (Kröger and Law, 2005). For instance, *Chlorella vulgaris* coupled to an optic fiber signal has been used to detect chlorophenols or pesticides (Védrine et al., 2003). Likewise, using this last immobilized whole-cell microalgae, a novel conductometric biosensor, based on alkaline phosphatase activity, has been designed to detect Cd^2+^ ions in aquatic habitats (Chouteau et al., 2004). For monitoring Cu^2+^ in reservoirs and water supplies the chlorophyta *Dictyosphaerium chlorelloides* has been used with an optic fiber coupled to a flow cell or a microwell-plate reader (Peña-Vázquez et al., 2010). Likewise, electrochemical (amperometric) biosensing systems for detecting toxic chemicals (toluene, Cu^2+^, or Ni^2+^) have been developed on the basis of motility of the flagellate microalga *Chlamydomonas reinhardtii* (Shitanda et al., 2005).

In many of these microorganisms, it is likely that the loss of usable genetic tools for bioengineering considerably limits the possible construction of WCBs; in fact, when they are used as bioreceptor elements in classic biosensors, they are usually wild type strains. There is, however, an exception related to the well-known microalgae model *C. reinhardtii*. In this unicellular alga, novel biorecognition elements suitable for herbicide sensing were obtained after mutation targeted at the photosystem-II D1 protein (Lambreva et al., 2013). Unfortunately, similar experiments using this more suitable microalga model have not yet been carried out for environmental heavy metal monitoring, even though studies have already been done on metal toxicity in this microorganism (Aksmann et al., 2014; De Schamphelaere et al., 2014). In the last one (De Schamphelaere et al., 2014), the authors compare Pb^2+^ sensitivity levels in three microalgae species (including *C. reinhardtii*). Aksmann et al. (2014) report an expression gene analysis of antioxidant enzymes: superoxide dismutase, catalase, and ascorbate peroxidase, under oxidative stress induced by Cd^2+^, together with an analysis of photosynthetic activity in this alga.

We therefore believe that this biotechnological aspect of microalgae has not been sufficiently exploited in view of the fact that they have enough qualities to be considered as good potential heavy metal WCBs.

## PROTOZOA AS HEAVY METAL WCBs

Among protozoa, ciliates have been extensively used in ecotoxicological studies (Gutiérrez et al., 2008). As well as all the previously mentioned, advantages of being a “eukaryotic microorganism,” ciliates also have at least, two additional advantages. Firstly, unlike bacteria, yeasts, or microalgae, ciliates are microorganisms without a cell wall in their vegetative stage. A major limitation to using microorganisms with cell walls as WCBs is the diffusion of substrates or molecules through the cell wall, resulting in a lower signal emission or less effective cell response. To prevent this, cells have to be permeabilized by physicochemical or enzymatic methods. The use of ciliates might therefore avoid or diminish this serious problem; the absence of a wall in these eukaryotic microorganisms results in greater sensitivity to environmental pollutants and a faster cell response (Martin-Gonzalez et al., 1999; Gutiérrez et al., 2003). Secondly, ciliates are eukaryotic cells with a series of metabolic traits that are more similar to those of human cells than bacteria, microalgae, or yeasts. Results obtained after completing genome sequencing projects in two ciliate models, *Tetrahymena thermophila* and *Paramecium tetraurelia* (Aury et al., 2006; Eisen et al., 2006), show that they share a higher degree of functional conservation with human genes than do other eukaryotic microbial models, such as yeasts; humans and *T. thermophila* share more ortholog genes with each other (about 2,280) than are shared between humans and *S. cerevisiae* (Eisen et al., 2006). It would therefore seem that this similarity with human biology makes it more reasonable to use them in ecotoxicological studies or as WCBs. Furthermore, ciliates are cosmopolitan microorganisms living in aquatic or terrestrial ecosystems, and can be used as WCBs for monitoring pollutants in both habitats. Likewise, these microorganisms have been widely used in heavy metal ecotoxicology (Gutiérrez et al., 2008, 2011).

*Tetrahymena thermophila* has five metallothionein (MT) gene isoforms. Two of these (*MTT1* and *MTT5*) are preferably over-expressed under Cd^2+^ or Pb^2+^ stress, respectively, though they are also induced by other metals (Díaz et al., 2007; Gutiérrez et al., 2011). Both genes, but mainly *MTT5*, respond quickly and strongly to metal stress, and their promoter regions are good candidate for designing heavy metal WCBs. The first two ciliate WCBs to detect metal environmental pollution were reported in 2011 (Amaro et al., 2011), using the *MTT1* or *MTT5* gene promoter from *T. thermophila* and luciferase as reporter gene. These transformed strains were used to design *turn on* bioassays to detect heavy metals in polluted soils and aquatic samples. Validation of these WCBs was carried out using artificial and natural (soil or aquatic) samples, including methods to detect false positives and negatives. A second type of *T. thermophila* WCB has been constructed with *MTT1* gene promoter and the GFP as the reporter molecule (Amaro et al., 2014). A comparative analysis of both types of WCBs reveals that: (i) the minimal exposure time to obtain a detectable signal is 1 h for luciferase WCB (MTT1Luc or MTT5Luc strains) and 2 h for GFP-WCB, indicating a faster response in those with luciferase as the reporter gene; (ii) the minimum detectable Cd^2+^ concentration is about 5–25 nM in luciferase WCBs, and 445 nM in GFP-WCBs, so luciferase WCBs are more sensitive than GFP-WCBs; (iii) the bioluminescence emission from luciferase WCB viable cells is up to 5 μM Cd^2+^, while cells with fluorescence emission (GFP-WCB) are viable up to 15 μM Cd^2+^. GFP-WCB cells are more resistant to Cd^2+^ than MTT5Luc or MTT1Luc strains, because they have a higher copy number (with plasmid constructs) of *MTT1* or *MTT5* genes (Amaro et al., 2014).

All these results indicate the great potential of ciliates as WCBs for monitoring metals in environmental polluted samples, and most probably other pollutants.

## A COMPARATIVE ANALYSIS

Although the three types of eukaryotic microorganisms (yeasts, microalgae, or ciliates) can be used as WCBs for heavy metal monitoring, they all have their advantages and disadvantages with respect to the others. The most significant are:

(i) Cell wall *versus* permeability. Unlike in ciliated protozoa, the presence of a cell wall in yeasts and microalgae may require a preliminary permeabilization process to facilitate the transit of the pollutant through the cell wall or membrane. This might disturb the response of the cell used as a WCB. Likewise, in order to obtain the reporter signaling in substrate-dependent reporters, the substrate must cross the cell wall and reach the cytoplasm, where the enzymatic reaction takes place, or be added to lysated cells. The substrate for eukaryotic luciferase, D-luciferin, is membrane-permeant only in its protonated form (pH 5); at neutral pH, it crosses the plasma membrane very slowly. For this reason, most luciferase-based bioassays are performed using cell extracts (Van der Meer et al., 2004) or with permeabilized cells (Lagido et al., 2001). As cell walls are absent in the vegetative phase, ciliates have a great advantage over other potential eukaryotic WCBs, because there is no need for any preliminary permeabilization treatment or cellular lysis. Permeabilization or cellular lysis is not necessary for *T. thermophila* used as a WCB with luciferase as the reporter gene, because the luciferin crosses through the cell pellicle. In this ciliate, luciferase activity can be measured as efficiently in intact viable cells as in permeabilized cells, and similar induction *in vivo* and *in vitro* is observed (Amaro et al., 2011). The efficient uptake of luciferin by *T. thermophila* makes this microorganism a more flexible WCB than other established eukaryotic biosensors.
(ii) Specificity *versus* sensitivity. The majority of WCBs respond to two or more metals, although some of them show greater specificity (Corbisier et al., 1999; Tom-Petersen et al., 2001; Ivask et al., 2009). Finding a gene promoter that responds exclusively to one metal is difficult, because of the complex cell interactions that take place during stress response, mainly in eukaryotic cells. Contaminated ecosystems are often polluted with a mixture of metals rather than a single one (Preston et al., 2000; Fairbrother et al., 2007). For this reason, one valuable aim of environmental biomonitoring may be to determine the overall toxicity of a sample rather than the specific metals present. Metal specificity is therefore not so important when designing a WCB to be used for monitoring environmental metal pollution. On the other hand, the sensitivity level of the WCB is of great importance when trying to detect metals present in very low concentrations, mainly those that are lower than the maximum allowable metal concentrations established for any ecosystem by international commissions. A comparative analysis of the ranking of sensitivity values to different heavy metals among reported eukaryotic WCBs shows that (a) with regard to the metaloid As^5+^, the *T. thermophila* MTT5Luc strain (with the reporter construct *MTT5::LucFF*) is the eukaryotic WCB with the highest sensitivity (25 nM; Amaro et al., 2011); (b) something similar occurs for Zn^2+^ (0.5 μM) in the *T. thermophila* MTT1Luc strain (with *MTT1::LucFF* construct), for Cd^2+^ (5 nM) in the *T. thermophila* MTT5Luc strain, for Hg^2+^ (0.25 nM) in the MTT1Luc strain and for Pb^2+^ (0.5 nM) in the MTT5Luc strain (Amaro et al., 2011); (c) with regard to Cu^2+^ ions, it is the *S. cerevisiae* WCB (strain with the *cup::gfp* construct) that has the greatest sensitivity to this metal (0.5 μM; Shetty et al., 2004). To sum up, among the eukaryotic microorganisms used as WCBs, ciliates are generally the most sensitive to heavy metals or respond to the lowest metal concentrations.

## CONCLUDING REMARKS

From this review on eukaryotic WCBs, the following general conclusions can be drawn:

(i) eukaryotic microorganisms used as WCBs have certain advantages over prokaryotic cells. Among them, extrapolation of the results to higher eukaryotic organisms is more reliable than using bacteria; (ii) inducible systems are more appropriate for designing heavy metal WCBs; (iii) the decision as to whether to use substrate-dependent or independent reporters will be determined by the greater or lesser capacity for permeability of the substrate through the wall or membrane of the cellular system used as WCB; (iv) in general, few WCBs are validated using bioassays with real environmental samples; (v) the biotechnology for using microalgae as WCBs is still underdeveloped, although these photosynthetic microorganisms have a great potential as biosensors based on genetic constructs involving photosynthesis genes; (vi) ciliates are eukaryotic microorganisms that have a series of advantages over yeasts or microalgae for designing heavy metal WCBs; and (vii) with regard to heavy metal WCBs for use in real environmental polluted samples, the capacity for sensitivity of the biosensor is more important than its level of specificity to a metal.

The future development of eukaryotic WCBs for environmental metal pollution monitoring could be considerably furthered by applying a synthetic biology approach. This would facilitate the design of WCBs with multi-input systems based on two or more regulatory gene promoters in the same gene construct, thereby increasing the capacity of the biosensor for detecting several different pollutants.

## AUTHOR CONTRIBUTIONS

The review was conceived and written equally by the three authors.

### CONFLICT OF INTEREST STATEMENT

The authors declare that the research was conducted in the absence of any commercial or financial relationships that could be construed as a potential conflict of interest.
